# Identification of a methylation profile for *DNMT1*-associated autosomal dominant cerebellar ataxia, deafness, and narcolepsy

**DOI:** 10.1186/s13148-016-0254-x

**Published:** 2016-09-05

**Authors:** Kristin D. Kernohan, Laila Cigana Schenkel, Lijia Huang, Amanda Smith, Guillaume Pare, Peter Ainsworth, Care4Rare Canada Consortium, Kym M. Boycott, Jodi Warman-Chardon, Bekim Sadikovic

**Affiliations:** 1 Children’s Hospital of Eastern Ontario Research Institute, University of Ottawa, 401 Smyth Road, Ottawa, Ontario K1H 8L1 Canada; 2 Department of Pathology and Lab Medicine, Western University, London, Ontario Canada; 3 Department of Pathology and Molecular Medicine, McMaster University, Hamilton, ON Canada; 4 Department of Clinical Epidemiology and Biostatistics, McMaster University, Hamilton, ON Canada; 5 Department of Biochemistry, Western University, London, ON Canada; 6 Children’s Health Research Institute, London, ON Canada; 7 Division of Neurology, The Ottawa Hospital, Ottawa, Ontario Canada; 8 Ottawa Hospital Research Institute, Ottawa, Ontario Canada; 9 Molecular Genetics Laboratory, Victoria Hospital, London Health Sciences Centre, 800 Commissioner’s Road E, London, ON N6A 5W9 Canada

**Keywords:** DNMT1, DNA methylation, CpG methylation array, Ataxia, Narcolepsy, Dementia, Hearing loss

## Abstract

**Background:**

DNA methylation is an essential epigenetic mark, controlled by DNA methyltransferase (DNMT) proteins, which regulates chromatin structure and gene expression throughout the genome. In this study, we describe a family with adult-onset autosomal dominant cerebellar ataxia with deafness and narcolepsy (ADCA-DN) caused by mutations in the maintenance methyltransferase DNMT1 and assess the DNA methylation profile of these individuals.

**Results:**

We report a family with six individuals affected with ADCA-DN; specifically, patients first developed hearing loss and ataxia, followed by narcolepsy, and cognitive decline. We identified a heterozygous *DNMT1* variant, c.1709C>T [p.Ala570Val] by Sanger sequencing, which had been previously reported as pathogenic for ADCA-DN and segregated with disease in the family. DNA methylation analysis by high-resolution genome-wide DNA methylation array identified a decrease in CpGs with 0–10 % methylation and 80–95 % methylation and a concomitant increase in sites with 10–30 % methylation and >95 % methylation. This pattern suggests an increase in methylation of normally unmethylated regions, such as promoters and CpG islands, as well as further methylation of highly methylated gene bodies and intergenic regions. Furthermore, a regional analysis identified 82 hypermethylated loci with consistent robust differences across ≥5 consecutive probes compared to our large reference cohort.

**Conclusions:**

This report identifies robust changes in the DNA methylation patterns in ADCA-DN patients, which is an important step towards elucidating disease pathogenesis.

**Electronic supplementary material:**

The online version of this article (doi:10.1186/s13148-016-0254-x) contains supplementary material, which is available to authorized users.

## Background

DNA methylation represses genomic expression through allosteric inhibition of transcription factors and transcriptional machinery, while serving as a recognition site for a host of repressive factors [1]. This mark is applied by the DNMT proteins: DNA methyltransferase 1 (DNMT1) is a maintenance methyltransferase, while DNMT3a and DNMT3b are de novo methyltransferases. There is a great deal of research aimed at understanding the role of DNA methylation in human health. To date, defects in DNA methylation have been identified as causative or contributing in numerous cancers, developmental syndromes, genetic disorders, and complex conditions (reviewed in [2–6]). Further research is required to fully understand the impact of altered methylation on human health and to utilize this knowledge for the development of effective therapeutics.

Autosomal dominant cerebellar ataxia with deafness and narcolepsy (ADCA-DN, OMIM 604121) is a degenerative disease caused by mutations in the DNA-targeting domain of the maintenance methyltransferase DNMT1 [7–9]. This adult-onset condition (onset in the second to fourth decade) is characterized by hearing loss, narcolepsy/cataplexy, and cerebellar ataxia; there are also reports of sensory neuropathy and psychiatric and behavioural manifestations. Interestingly, a second condition, hereditary sensory neuropathy with dementia and hearing loss (HSAN1E), is also caused by mutations in *DNMT1*. While once classified as distinct disorders, we now recognize that HSAN1E and ADCA-DN patients demonstrate significant overlap in clinical features, suggesting that these are likely the same condition with clinical variability depending on location and type of mutation and an individual’s genetic background [10]. Molecular profiling of HSAN1E patient cells has uncovered both global DNA hypomethylation and site-specific hypermethylation [11, 12]. It remains to be seen if these methylation changes are consistent or different between HSAN1E and ADCA-DN and how they might contribute to the aforementioned phenotypes. Additionally, further studies are required to inform the phenotypic spectrum of this condition.

## Methods

### Clinical specimens

A family with six affected individuals presented to the Medical Genetics Service and was enrolled in the Care4Rare Canada study due to the absence of a molecular diagnosis. Approval of the study design was obtained from the institutional research ethics board (Children’s Hospital of Eastern Ontario) and informed consent obtained from all participants.

### Methylation array

DNA methylation array was performed on blood DNA using the Infinium HumanMethylation450 BeadChip (Illumina) on five individuals according to standard protocol (Genetic and Molecular Epidemiology Laboratory, McMaster University). Methylation data (beta value) was converted to .idat files using the Genome Studio Software and imported into the Partek Genomics Suite (PGS) software for statistical analysis. Statistical analyses were performed using PGS, comparing the patient cohort (5 individuals) with 361 normal controls (reference cohort). Our controls included individuals that were previously preselected from a larger cohort of about 1000 individuals across the broad range of age, sex, and ethnicity distribution. The methylation analysis of these individuals was performed in the same facility as patients, and the same data processing pipeline was used. Based on the individual analysis (1 sample versus cohort), these reference controls showed no significant changes in DNA methylation relative to the entire reference cohort. This analysis takes into account the fact that significant portion of genomic DNA methylation is hyper-variable across individuals (including age-related hyper-variable regions). Such regions with the normal inter-individual and/or age-related methylation variability would not produce significant *p* values when comparing an individual or a patient cohort to a reference. Data analysis was performed using modifications of the protocol previously described by Schenkel et al. [13]. Briefly, an ANOVA test was performed to generate probe-level statistics and regions with significant DNA methylation patterns which had (1) a minimum of five consecutive probes with probe-level *p* < 0.01, (2) mean *F*-value across the region >50, and (3) estimate value (methylation difference between groups) higher than 0.20 (+/−). This approach focuses on identification of genomic regions with significant methylation differences encompassing multiple immediately adjacent probe sets using multilayered statistical criteria including the *p* value, *F*-value, and estimate (mean methylation difference) across a region encompassing at least five contiguous probes. Significant regions were mapped against the CpG islands and gene promoter regions using human reference genome Hg19. Data was visualized using the PGS genomic browser.

### Pathway analysis

The top differentially methylated genes identified were assessed using the pathway analysis tool in the PGS. Briefly, statistical analysis included Fisher exact test and restriction to functional groups containing at least two genes. Results show the enrichment *p* value (*p* value of the Fisher exact test reflective of the number of the genes in versus not in the list or functional group) and the enrichment score (ES; negative log of the enrichment *p* value; a high score indicates that the genes in the functional group are overrepresented in the gene list).

### Bisulfite mutagenesis

Genomic DNA isolated from blood of patients and age-matched controls was sodium bisulfite treated using the EZ DNA Methylation-Direct Kit (Zymo Research) according to the manufacturer’s instructions. DNA was amplified by nested PCR and the resulting products ligated into the pCR2.1 vector using a TOPO-TA cloning kit (Invitrogen). Positive clones were sequenced with Applied Biosystem 3730xl DNA Analyzer technology (Centre for Applied Genomics, McGill University). Clones were accepted at ≥95 % conversion. Non-converted cytosine residues and mismatched base pairs were used to ensure all clones originated from unique template DNA.

## Results

### Family description

A family of six affected individuals presented to the Genetics Service at the Children’s Hospital of Eastern Ontario over two decades. Individuals demonstrated a wide phenotypic spectrum; prominent clinical symptoms included progressive narcolepsy, length-dependent sensory neuropathy, ataxia, optic atrophy with cognitive decline, and personality changes such as impulsivity. Symptoms began in the second to fourth decade of life. Additionally, most members presented with hearing loss in the third and fourth decades. Marked lymphedema and urinary urgency were also prominent in the three female patients.

These patients were recognized as sharing significant clinical features with those described by Winkelman et al. with DNMT1 mutations [7]. As such, we sequenced the DNMT1 gene and identified a variant, NM_001130823.1:c.1709C>T [p.Ala570Val], which was one of the originally reported mutations [7]. This variant segregated with the disease within the family. These findings led us to conclude that this heterozygous variant is likely disease-causing and to further investigate the molecular phenotype of these patients.

### Identification of a unique DNA methylation signature for ADCA-DN

**Fig. 1:**
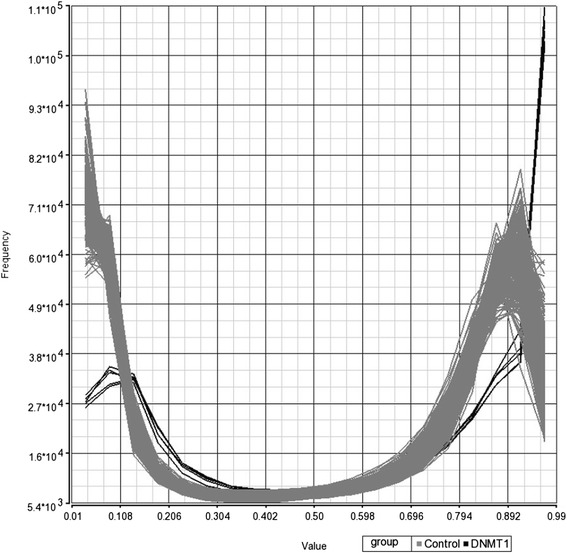
Histogram of genome-wide DNA methylation array data showing frequency of methylation levels across the genome in patients with ADCA-DN (*black*) and controls (*grey*). Patients with ADCA-DN showed a lower frequency of CpGs with 0–10 % methylation and 80–95 % methylation and a concomitant increase in sites with 10–30 % methylation and >95 % methylation. This pattern suggests an increase in methylation of normally unmethylated regions, such as promoters and CpG islands, as well as further methylation of normally hypermethylated gene bodies and intergenic regions

Given the well-documented function of DNMT1, combined with previous cellular phenotyping descriptions in HSAN1E patients [11, 12], we chose to investigate global DNA methylation signatures in this ADCA-DN family. We performed a genome-wide assessment of DNA methylation using the high-resolution Infinium HumanMethylation450 BeadChip, which assesses 480,000 methylation sites across unique (non-repetitive) DNA sequences, including 96 % of CpG islands and 99 % of RefSeq genes, with an average of 17 CpGs per gene. This analysis identified global methylation changes in the patients relative to controls (Fig. 1). Specifically, there was a decrease in hypomethylated probes (0–10 % methylation) and an increase in probes with 10–30 % methylation. This pattern suggests increased methylation in normally unmethylated regions, most of which are located in promoters and CpG islands. We also identified a number of hypermethylated regions, signified by a decrease in probes with 80–95 % methylation and an increase in probes with >95 % methylation. Hypermethylation normally occurs in gene bodies and intergenic regions, suggesting further methylation of these sites. A regional analysis (≥5 consecutive probes) revealed 82 regions with significant methylation changes. All of these sites were hypermethylated in ADCA-DN patients and were distributed across different chromosomes. Of these 82 regions, 45 overlapped CpG islands in gene promoters, 20 were located in CpG islands outside of genes, 11 were located in CpG shores or shelves, and 6 were located in intergenic non-CpG island locations (Additional file 1: Table S1).

Many of the genes identified in the methylation array are associated with important developmental process, and disruption of their expression by altered DNA methylation may play a role in disease pathogenesis. Pathway analysis of the 82 differentially methylated regions revealed an enrichment for cellular developmental processes (including 30 genes, ES = 24, *p* < 0.01) and anatomical structure development (including 29 genes, ES = 20, *p* < 0.01).

**Fig. 2:**
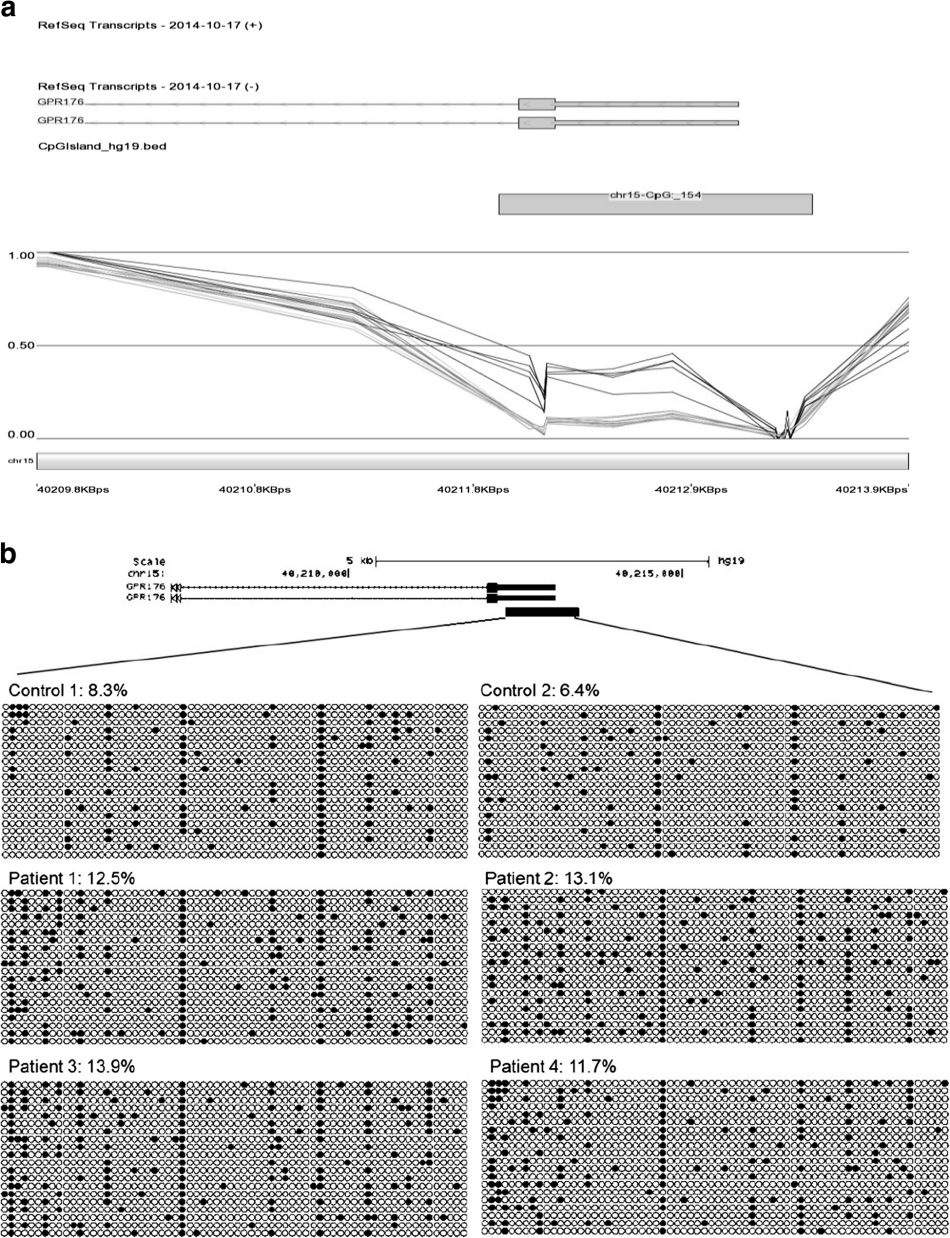
**a** Methylation visualization of significantly altered gene *GPR176* in ADCA-DN patients (*black*) and controls (*grey*) identified by methylation array. Figures were generated using genomic browser viewer (Partek) and show methylation levels 0 (not methylated) and 1 (100 % methylated) among controls and ADCA-DN patients. Location of CpG islands and genes are also represented. **b** Bisulfite mutagenesis and sequencing analysis of the *GPR176* promoter region confirms effects seen by methylation array analysis. Twenty alleles from each sample were analysed, and individual alleles are represented as a string of CpGs. The total percent methylation for each sample is indicated. Unmethylated CpGs are represented as *empty circles* and methylated CpGs as *filled circles*

To confirm our methylation array findings, we performed bisulfite mutagenesis and sequencing at the *GPR176* promoter region (Fig. 2). Methylation array at this region showed hypermethylation in ADCA-DN patients (Fig. 2), with average methylation of five adjacent probes at 8 % and 32 % in controls and patients, respectively. Using bisulfite mutagenesis, we detected an overall 1.75-fold increase in methylation of this sequence (controls 7.35 % versus patients 12.83 %). These findings confirm the accuracy and specificity of the DNA methylation array data.

## Discussion

Previous studies have identified *DNMT1* mutations in ADCA-DN and HSAN1E. While once described as distinct disorders, these are now recognized as the same condition which presents along a clinical spectrum [10]. In this study, the family’s presentation is most similar to the ADCA-DN end of the spectrum. Mounting evidence highlights the importance of DNA methylation for proper neurological development and function. For example, changes in the methylome have been identified in conditions such as brain cancer, autism, intellectual disability syndromes, Alzheimer and Parkinson diseases, amyotrophic lateral sclerosis, epilepsy, multiple sclerosis, and now ADCA-DN/HSAN1E [3–5, 7]. While these conditions differ greatly in their clinical presentations, including central and peripheral nervous system involvement, they highlight the importance of the neuronal methylome.

Using the Infinium HumanMethylation450 array platform, we identified increased methylation in normally unmethylated promoters and CpG islands, as well as further methylation of hypermethylated gene bodies and intergenic regions in ADCA-DN patients. These findings are highly similar to those of previous studies on HSAN1E patients which have demonstrated hypermethylated regions using low-resolution Infinium HumanMethylation27 arrays, global hypomethylation using non-site-specific liquid chromatography-electrospray ionization tandem mass spectrometry, and specific sites of hypomethylation and hypermethylation at a very high resolution by genome-wide bisulfite sequencing [11, 12]. Note that mass spectrometry analysis would include repetitive elements which carry a significant portion of genomic methylation, while methylation arrays and bisulfite sequencing are restricted to unique sequences and thus do not assess global methylation status. Taken together, it is likely that mutations in *DNMT1* cause similar hypomethylation at repetitive elements and hypermethylation at specific gene/promoter/CpG islands in both HSAN1E and ADCA-DN. Importantly, we identified 82 hypermethylated regions with robust methylation changes in at least five consecutive probes and propose that the methylation profile of these 82 regions should be further investigated with regard to implications for disease pathogenesis.

## Conclusions

Overall, our methylation findings expand the understanding of ADCA-DN molecular pathogenesis a critical first step on the long journey to development of an effective therapy. While there are phenotypic differences throughout the spectrum of HSAN1E and ADCA-DN patients, we observe the same general methylation trends. Further study is required to elucidate the underlying disease mechanisms and to determine if and how these methylation changes contribute to disease pathogenesis.

## Additional file

Additional file 1: Significant regions detected by methylation array in ADCA-DN versus control individuals.

## Abbreviations

DNMT1: DNA methyltransferase 1
ADCA-DN: Autosomal dominant cerebellar ataxia with deafness and narcolepsy
HSAN1E: Hereditary sensory neuropathy with dementia and hearing loss
CpGs: CpG dinucleotides
