# Supplementary material for: Identification of a methylation profile for DNMT1-associated autosomal dominant cerebellar ataxia, deafness, and narcolepsy
Source: Clin Epigenetics. 2016 Sep 5;8(1):91. doi: 10.1186/s13148-016-0254-x (PMC5011850; doi:10.1186/s13148-016-0254-x)
Supplement: Additional file 1: — Significant regions detected by methylation array in ADCA-DN versus control individuals. [file 13148_2016_254_MOESM1_ESM.pdf]

Supplementary Table 1: Significant regions detected by methylation array in ADCA-DN versus control individuals

Chr	Region start	Region end	Meth. average	# probes	Estimate	Nearest gene	Distance to nearest gene	Distance to nearest CpG island
chr16	67223723	67224639	0.751	5	0.22	EXOC3L1 (-)	0	4503
chr3	46618658	46619374	0.659	5	0.23	TDGF1 (+)	0	0
chr3	16216084	16216616	0.639	5	0.20	GALNT15 (+)	0	89521
chr15	29213240	29213870	0.600	7	0.21	APBA2 (+)	0	81031
chr7	150885074	150885664	0.599	5	0.20	ASB10 (-)	154	13037
chr14	94461903	94462617	0.591	5	0.20	LINC00521 (+)	999	30023
chr6	32158917	32159617	0.526	6	0.22	GPSM3 (-)	0	3676
chr6	30853245	30853618	0.500	5	0.25	DDR1 (+)	0	568
chr6	28555643	28555939	0.492	5	0.22	ZBED9 (-)	530	518
chr6	33240458	33241036	0.458	5	0.30	RPS18 (+)	0	358
chr12	6745047	6745578	0.420	6	0.21	LPAR5 (-)	0	10424
chr11	32449153	32450114	0.367	7	0.29	WT1 (-)	0	0
chr17	21355997	21356382	0.365	5	0.26	KCNJ18 (+)	35514	0
chr2	19553227	19553960	0.361	5	0.22	OSR1 (-)	0	0
chr5	134872114	134872669	0.303	8	0.21	NEUROG1 (-)	474	62
chr8	48099605	48100196	0.300	5	0.22	LOC100287846 (+)	734	0
chr2	39187523	39188016	0.297	6	0.20	ARHGEF33 (+)	0	0
chr5	2749705	2750768	0.295	5	0.23	IRX2 (-)	0	0
chr19	18343292	18344332	0.279	5	0.22	PDE4C (-)	0	0

chr4	298671	299380	0.279	9	0.22	ZNF732 (-)	8726	0
chr1	47898901	47899686	0.275	5	0.23	FOXD2-AS1 (-)	0	0
chr2	132182294	132183118	0.250	5	0.20	LINC01120 (+)	16492	0
chr19	52390800	52391377	0.237	10	0.34	ZNF649-AS1 (+)	0	0
chr5	140419809	140420528	0.222	6	0.30	LOC101926905 (-)	5262	55072
chr3	153840099	153840728	0.218	8	0.23	ARHGEF26 (+)	0	0
chr2	177003396	177004025	0.216	5	0.25	HOXD-AS2 (-)	1569	428
chr1	75593794	75594271	0.214	6	0.21	LHX8 (+)	0	1543
chr6	137813574	137814366	0.213	5	0.22	OLIG3 (-)	0	0
chr4	174442533	174443376	0.209	5	0.20	HAND2 (-)	4276	0
chr4	5894681	5895420	0.207	5	0.28	CRMP1 (-)	0	0
chr13	36048882	36050168	0.203	6	0.25	MIR548F5 (-)	0	0
chr6	100903681	100904386	0.199	7	0.20	SIM1 (-)	0	0
chr14	57276779	57277771	0.198	6	0.24	OTX2 (-)	0	0
chr4	54974634	54975687	0.198	7	0.20	GSX2 (+)	6511	0
chr5	115297680	115298730	0.195	9	0.24	AQPEP (+)	0	0
chr7	155301724	155303308	0.191	7	0.30	CNPY1 (-)	0	0
chr5	122430225	122430831	0.184	7	0.24	PRDM6 (+)	0	0
chr1	236849379	236850242	0.182	7	0.25	ACTN2 (+)	0	0
chr5	87968108	87968668	0.182	5	0.20	LINC00461 (-)	0	0
chr2	162273427	162274809	0.178	7	0.25	TBR1 (+)	0	0
chr19	50706473	50706884	0.176	6	0.24	MYH14 (+)	1	0

chr5	72598855	72599796	0.171	6	0.24	TMEM174 (+)	127884	0
chr4	187646354	187646939	0.170	5	0.24	FAT1 (-)	1366	0
chr4	113441693	113442429	0.168	5	0.23	NEUROG2 (-)	4364	1753
chr2	223176596	223177018	0.166	5	0.20	CCDC140 (+)	6659	0
chr13	112716613	112717254	0.163	5	0.22	LINC00403 (+)	0	0
chr3	147126111	147126971	0.163	10	0.23	ZIC1 (+)	210	18
chr11	88241584	88242498	0.159	5	0.22	GRM5-AS1 (+)	0	0
chr7	97360605	97361211	0.156	5	0.27	TAC1 (+)	60	0
chr1	50890143	50891205	0.155	6	0.23	DMRTA2 (-)	1023	0
chr1	91182205	91182866	0.145	8	0.22	BARHL2 (-)	0	0
chr12	63543821	63544867	0.144	5	0.22	AVPR1A (-)	0	0
chr18	5197378	5197569	0.144	5	0.21	C18orf42 (-)	122	0
chr2	175193367	175194089	0.143	6	0.20	LINC01305 (+)	0	0
chr12	22094553	22095192	0.142	6	0.24	ABCC9 (-)	4924	0
chr3	147127183	147129288	0.142	11	0.21	ZIC1 (+)	0	0
chr13	112720917	112723591	0.136	18	0.23	SOX1 (+)	0	0
chr17	47075165	47075890	0.133	5	0.21	IGF2BP1 (+)	0	0
chr6	106428947	106429669	0.130	8	0.25	PRDM1 (+)	104526	0
chr6	6004454	6005220	0.128	6	0.21	NRN1 (-)	0	0
chr13	79169704	79171479	0.127	15	0.22	RNF219-AS1 (+)	0	0
chr12	103358609	103358891	0.127	6	0.21	ASCL1 (+)	4314	358
chr1	53308587	53309271	0.125	6	0.28	ZYG11A (+)	0	0

chr2	162279954	162281121	0.123	6	0.23	TBR1 (+)	0	0
chr2	227700104	227700578	0.119	7	0.26	RHBDD1 (+)	93	0
chr1	197887285	197888315	0.118	8	0.20	LHX9 (+)	0	0
chr8	145910536	145911670	0.112	8	0.21	ZNF251 (-)	34624	0
chr3	27765222	27765556	0.110	5	0.21	EOMES (-)	1015	0
chr5	16179002	16179670	0.109	5	0.25	MARCH11 (-)	0	0
chr14	29235894	29237490	0.107	16	0.25	FOXG1 (+)	0	0
chr10	22634028	22634612	0.107	11	0.21	SPAG6 (+)	0	0
chr5	112823161	112824298	0.101	5	0.25	MCC (-)	0	0
chr15	40212096	40212791	0.096	5	0.24	GPR176 (-)	0	0
chr8	24771115	24771655	0.096	6	0.25	NEFM (+)	0	0
chr5	72715454	72716132	0.095	7	0.25	FOXD1 (-)	25953	0
chr6	100912812	100913602	0.093	7	0.20	SIM1 (-)	1260	0
chr13	25745396	25745996	0.091	5	0.26	AMER2 (-)	0	0
chr15	30114285	30115309	0.086	10	0.25	TJP1 (-)	0	0
chr11	2291250	2292014	0.081	8	0.23	ASCL2 (-)	0	0
chr5	42951701	42952379	0.074	5	0.24	FLJ32255 (-)	33122	0
chr20	21377661	21378237	0.059	8	0.20	NKX2-4 (-)	0	0
chr1	47882255	47882749	0.031	5	0.20	FOXE3 (+)	0	0

Human Reference Genome: hg19; Estimate value represents extend of methylation change (methylation difference between groups); Nearest gene shows 5’-3’ (+) and 3’-5’ (-) strands; Distance to nearest feature is presented in bps; Significant regions: Probes>5, Estimate > 20%, *p*-value<0.05
